# Supplementary material for: Reduced form of Galectin-1 Suppresses Osteoclastic Differentiation of Human Peripheral Blood Mononuclear Cells and Murine RAW264 Cells In Vitro
Source: Biomolecules. 2024 Jan 17;14(1):121. doi: 10.3390/biom14010121 (PMC10813495; doi:10.3390/biom14010121)
Supplement: Supplementary file 1 [file biomolecules-14-00121-s001.zip › biomolecules-2770108-supplementary.pdf]

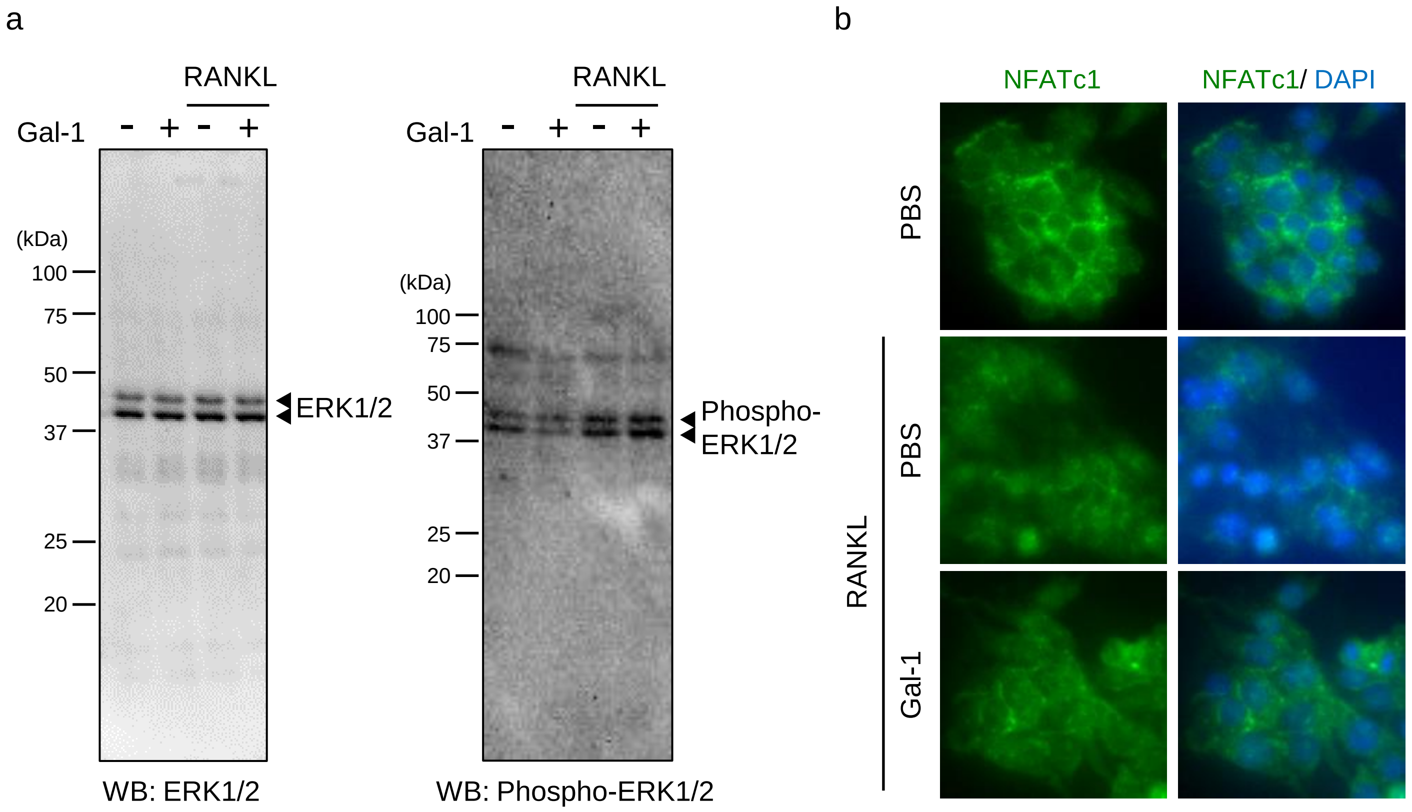


**Figure S1. Recombinant Gal-1 protein have little effect on early phase of osteoclast differentiation signal of mouse RAW264 cells**. **(a)** RAW264 cells were seeded in a 6 well cell culture plate at a density of 1 x $10^6$ cells/well and cultured for 1 day. Then the cells were pretreated with recombinant mouse Gal-1C2S (10 µg/mL) for 2 h, followed by the stimulation with sRANKL (250 ng/mL). After 15 min, the cells were harvested and subjected to western blotting using anti-ERK1/2 antibody (Cell signaling technology; #4695) or anti-phospho-ERK1/2 antibody (Cell signaling technology; #9101). **(b)** RAW264 cells were seeded in a 4 well chamber slide at a density of 2.5 x $10^3$ cells/well and cultured for 1 day. Then the cells were treated with mouse Gal-1C2S (10 µg/mL) and sRANKL (250 ng/mL) and cultured for 24 h. Then the cells were subjected to immuno-staining using anti-NFATc1 antibody (Cell signaling technology; #8032) and Alexa488-labeled anti-rabbit IgG secondary antibody (Thermo Fisher Scientific; #A11008). Nuclei were stained with VECTA SHIELD Mounting Medium with DAPI (Funakoshi; #H-1200). Fluorescently sained cells were observed and visualized using the BZ-X800 fluorescence microscope (Keyence) with a 20x objective lens.
